# Algorithmovigilance in AI-Based Oral-Health Surveillance: Temporal Drift, Cross-Survey Differences, and Predictor-Level Structural Stability in Population-Level Severe Tooth Loss Prediction

**DOI:** 10.3390/diagnostics16162569

**Published:** 2026-08-14

**Authors:** Quang Tuan Lam, Fang-Yu Fan, Yung-Li Wang, Sheng-Wei Feng, Thi Thuy Tien Vo, Minh Huu Nhat Le, Giang Vu, Nguyen Quoc Khanh Le, I-Ta Lee

**Affiliations:** 1 School of Dentistry, College of Oral Medicine, Taipei Medical University, 250 Wuxing St., Taipei 11031, Taiwan; d204111005@tmu.edu.tw (Q.T.L.); cetuswang@tmu.edu.tw (Y.-L.W.); shengwei@tmu.edu.tw (S.-W.F.); 2 AIBioMed Research Group, Taipei Medical University, 250 Wuxing St., Taipei 11031, Taiwan; 3 Faculty of Odonto-Stomatology, Da Nang University of Medical Technology and Pharmacy, Da Nang 50000, Vietnam; 4 School of Dental Technology, College of Oral Medicine, Taipei Medical University, Taipei 11031, Taiwan; fish884027@tmu.edu.tw; 5 Faculty of Dentistry, Nguyen Tat Thanh University, Ho Chi Minh City 70000, Vietnam; vtttien@ntt.edu.vn; 6 Section of Cardiovascular Medicine, Department of Internal Medicine, Yale School of Medicine, New Haven, CT 06510, USA; d142111009@tmu.edu.tw; 7 School of Global Health Management and Informatics, University of Central Florida, Orlando, FL 32816, USA; giang.vu@ucf.edu; 8 Artificial Intelligence in Medicine, College of Medicine, Taipei Medical University, Taipei 11031, Taiwan; 9 Translational Imaging Research Center, Taipei Medical University Hospital, Taipei 11031, Taiwan

**Keywords:** artificial intelligence, oral health, severe tooth loss, algorithmovigilance, temporal drift

## Abstract

**Background/Objectives**: Artificial intelligence models may retain acceptable discrimination while calibration and predictor–risk relationships change across populations and data-collection systems. We evaluated a structured algorithmovigilance framework for detecting temporal drift, cross-survey differences, and predictor-level structural instability in severe tooth loss prediction. **Methods**: Across five BRFSS cycles from 2016 to 2024 (total N = 2,176,039), a survey-weighted main-effects Explainable Boosting Machine trained in 2016 was evaluated chronologically through 2024. NHANES 2015–2018 served as an external reference for an exploratory cross-survey temporal comparison. **Results**: AUC declined modestly from 0.8638 in 2016 to 0.8495 in 2024. The frozen unrecalibrated model yielded AUC = 0.8681 and Brier Score = 0.1131 in pooled NHANES. The survey-system-by-period interaction was positive (beta = 0.0412; HC1 *p* = 0.014), although a stratified survey-weighted bootstrap sensitivity produced a wider interval including zero (95% CI, −0.0085 to 0.0895; *p* = 0.093). Smoking (*p* < 0.001) and income (*p* = 0.030) showed nominal predictor-level structural shifts, whereas BMI did not. An interaction-enabled EBM improved AUC by 0.0010 in 2016 and 0.0016 in 2024. **Conclusions**: Aggregate discrimination alone did not capture calibration and predictor-level changes. The framework provides retrospective monitoring signals for governed review, not evidence of causal survey-mode effects, formal algorithmic fairness, or clinical deployment readiness.

## 1. Introduction

The integration of machine learning (ML) into oral-health prediction and population-level risk surveillance has created new opportunities for large-scale surveillance, prevention-oriented screening, and risk monitoring [1,2]. Predictive algorithms trained on national health databases have shown utility for modeling complex oral-health outcomes, including severe tooth loss (STL), commonly defined as the loss of six or more permanent teeth [3]. STL is a marker of impaired oral function and a clinically meaningful cumulative oral-health outcome. This specific outcome was selected for evaluating algorithmic drift for several methodological reasons: (1) it is strongly socially patterned and associated with income, education, and healthcare access, making it suitable for predictor-level structural-stability analysis; (2) STL prevalence has been declining over time in the U.S., providing a natural temporal gradient; (3) both self-reported (BRFSS) and clinically examined (NHANES) STL data are available, enabling cross-system comparison; and (4) it is a cumulative, irreversible outcome integrating long-term exposure to multiple risk factors. However, using STL as a monitoring indicator also presents inherent limitations. As a cumulative outcome, it may respond slowly to recent environmental or behavioral changes, potentially attenuating the sensitivity of early drift detection. Furthermore, STL is an etiologically heterogeneous endpoint that conflates distinct clinical pathways, such as periodontal disease, severe dental caries, and trauma.

Recent applications of artificial intelligence in dentistry encompass caries detection, periodontal disease classification, orthodontic planning, and oral cancer screening [4,5,6,7,8]. Many population-level prediction models are nevertheless developed and validated in static datasets, with AUC emphasized more often than temporal calibration and transportability [9,10]. A model may therefore retain acceptable aggregate discrimination while its calibration or predictor–risk relationships change [11,12]. Here, algorithmovigilance denotes a governance-oriented process that links continuing assessment of discrimination, calibration, transportability, and structural stability to human review [13]. It extends routine technical monitoring and complements regulatory post-market surveillance. Recent clinical-AI monitoring research likewise emphasizes that performance signals must be connected to multidisciplinary review and operational response [14,15].

Regulatory and governance frameworks increasingly emphasize lifecycle monitoring, including the FDA AI/ML-Based Software as a Medical Device action plan [16], WHO guidance [17], and the EU AI Act [18]. Practical monitoring workflows for population-level dental prediction remain limited. This gap matters because models used for prevention planning or risk stratification may drift without an obvious failure in aggregate AUC. The present study therefore evaluates retrospective monitoring signals rather than deployment readiness.

Interpretability is also central to longitudinal auditing. Post hoc explanations may be vulnerable to perturbation or manipulation [19], whereas Explainable Boosting Machines (EBMs) provide model-intrinsic shape functions that can be compared across cohorts [20,21]. Main-effects EBMs do not capture pairwise interactions, creating a potential predictive trade-off. Contemporary Transformer-based tabular foundation models, including TabPFN, provide an additional high-performance comparison class [22], but they do not yield the same directly comparable univariate audit estimand. We therefore retained the main-effects EBM for the primary structural analysis and evaluated an interaction-enabled EBM as a targeted sensitivity analysis.

**Figure 1:**
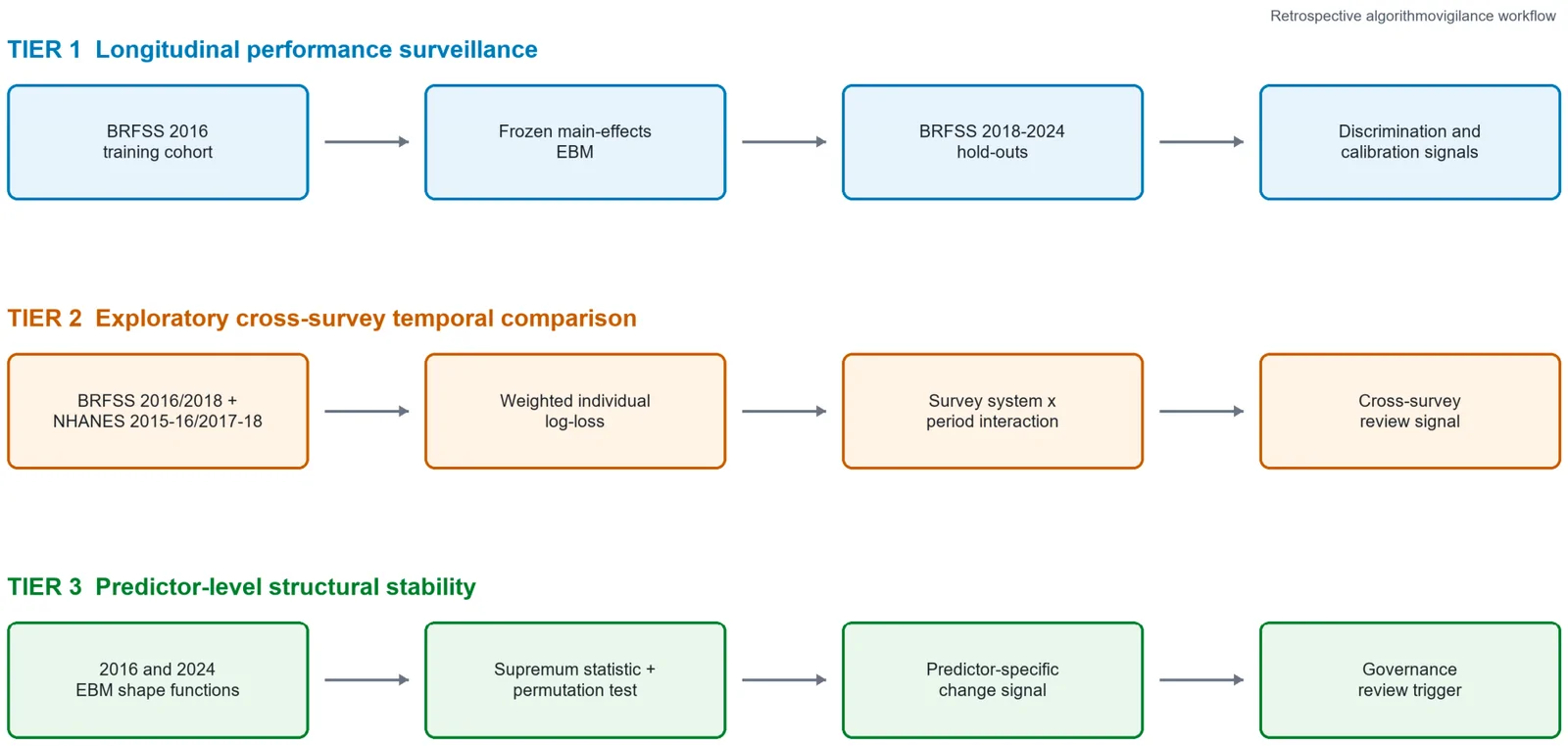
Three-tier retrospective algorithmovigilance framework. Tier 1 evaluates longitudinal discrimination and calibration of the frozen 2016 EBM across BRFSS hold-outs. Tier 2 compares observed BRFSS and NHANES periods using survey-weighted individual log-loss and an exploratory survey-system-by-period interaction. Tier 3 evaluates predictor-level structural stability by comparing EBM shape functions. The resulting quantities are monitoring signals for governed review rather than causal decompositions or formal fairness metrics.

The aim was to determine whether a longitudinal algorithmovigilance framework could detect temporal drift, cross-survey differences, and predictor-level structural instability not captured by aggregate discrimination alone. The three monitoring components were: (1) calibration surveillance across BRFSS 2016–2024; (2) an exploratory BRFSS–NHANES survey-system-by-period comparison; and (3) shape-function permutation testing for selected predictors. The model uses survey-derived sociodemographic, behavioral, and systemic-health predictors and is intended as a population-level risk-classification use case, not a chairside diagnostic or incident-risk model. Its contribution is a retrospective qualification workflow for future local and prospective validation. The framework is summarized in Figure 1.

## 2. Materials and Methods

This study was reported in accordance with the TRIPOD+AI guidelines [23] (Supplementary Table S1). All analytical procedures and temporal isolation protocols were designed to prevent retrospective data leakage and to emulate prospective validation of an AI-based oral-health risk prediction model across an eight-year surveillance horizon.

### 2.1. Dataset, Study Population, and Variable Operationalisation

The primary data source was the Behavioral Risk Factor Surveillance System (BRFSS). We aggregated five independent biennial cycles from 2016 to 2024 [24,25,26,27,28], yielding a total analytic sample of 2,176,039 respondents. This timeframe captured the pre-pandemic baseline, the COVID-19 disruption period, and an important shift in BRFSS survey administration. In 2016, wireless-only households surpassed 50% in the United States [29], providing relevant context for longitudinal changes in BRFSS survey administration.

The external reference data comprised NHANES 2015–2016 (n = 5460) and 2017–2018 (n = 5315), for a total of 10,775 participants [30,31]. These two observed periods were used in the exploratory cross-survey temporal comparison. NHANES is a methodologically independent reference, not an exchangeable control population or clinical gold standard. BRFSS used self-reported tooth loss, whereas NHANES used examination data and excluded third molars; these differences constrain all absolute cross-survey performance and calibration comparisons.

The target variable was STL, operationally defined as loss of six or more permanent teeth. This cutpoint corresponds to the CDC oral-health surveillance indicator for adults aged 65 years and older [32]; in this study, the same count threshold was applied across the analytic age range to define the binary outcome. This threshold is distinct from complete tooth loss (edentulism), which refers to loss of all natural teeth; edentulism was not the outcome of interest. In BRFSS, STL was defined using the self-reported variable _RMVTETH. In NHANES, tooth loss was quantified objectively using dental examination data (OHX). Third molars (positions 1, 16, 17, and 32) were excluded from the NHANES tooth count to reduce confounding from prophylactic or orthodontic extractions and congenital agenesis. This exclusion could not be applied to BRFSS because self-reported tooth loss does not identify specific tooth positions. This asymmetry is a consequence of the different measurement modalities and introduces a potential source of misalignment between the two outcome definitions. Respondents with missing outcome data were excluded.

The predictor set comprised 19 sociodemographic, behavioral, and systemic-health features relevant to population-level oral-health risk prediction. All predictors were verified as concurrent point-in-time variables to prevent look-ahead bias and were harmonized across datasets using unified categorical encoding. Complex survey design elements, including primary sampling units, strata, and sampling weights, were retained to preserve nationally representative estimation. Because this study used exclusively de-identified, publicly available CDC datasets, it was exempt from Institutional Review Board review under 45 CFR Part 46. Biological sex was included as a predictor variable in accordance with SAGER guidelines. Sex-stratified subgroup analysis was beyond the primary scope of this study but represents an important direction for future formal subgroup assessment of AI-based oral-health risk prediction models (Table 1).

### 2.2. Missing Data Imputation and Explainable Boosting Machine

Missing data, the pre-imputation proportions of which are detailed for each variable in Table 1, were addressed using Multiple Imputation by Chained Equations (MICE) [33] under a zero-leakage protocol. Specifically, five imputed datasets were generated per cycle. The imputation process utilized predictive mean matching for continuous variables, logistic regression for binary variables, and polytomous regression for multi-category variables, with 20 iterations per chain to ensure convergence. Convergence was assessed using trace plots and distributional comparisons. To prevent retrospective data leakage, imputation models were fitted separately within each cycle’s training subset and then applied only to the corresponding hold-out test subset. Pooled estimates for subsequent evaluations were calculated using Rubin’s rules. This approach ensured that information from future cycles or test data were not used during model development or preprocessing.

Predictive modeling used a survey-weighted EBM restricted to main effects [34,35]. This specification preserves directly comparable univariate shape functions across temporal cohorts. Because disabling interactions may reduce predictive performance, a targeted sensitivity analysis fitted an otherwise like-for-like interaction-enabled EBM to the same 2016 training cohort and evaluated it on the 2016 and 2024 hold-outs.

The 2016 BRFSS cohort was split 80/20 into a survey-weighted training set (N = 379,903) and hold-out set (N = 94,976) using random state 42 and outcome stratification. The main-effects Explainable Boosting Classifier used interactions = 0, learning rate = 0.01, 1024 bins, 14 outer bags, 0 inner bags, a 15% internal early-stopping split, 50-round patience, and a maximum of 25,000 boosting rounds. Chronological BRFSS hold-outs were not used for model selection. The interaction sensitivity cloned these settings except for automatic pairwise interaction selection (interactions = 0.9; 18 selected terms). Supplementary Table S2 provides the configuration, and Supplementary Table S11 reports the interaction sensitivity.

### 2.3. Analytical Framework and Statistical Evaluation

The framework evaluated global model performance, calibration stability, exploratory cross-survey differences, and predictor-level structural stability. Performance metrics used cycle-specific normalized survey weights. All tests were two-sided at alpha = 0.05. The original individual-level WLS comparison used HC1 heteroskedasticity-consistent standard errors; HC1 does not explicitly account for PSU clustering or stratification. Robustness was therefore examined post hoc using a 2000-replicate stratified survey-weighted bootstrap with survey-cycle-specific identifiers. The retained BRFSS PSU field was effectively respondent-level within strata, so the BRFSS component represents stratified respondent resampling; NHANES retained genuine PSU-within-stratum resampling.

#### 2.3.1. Temporal Drift Evaluation

The 2016 baseline model was chronologically evaluated using hold-out BRFSS cohorts from 2018 to 2024. Model performance was assessed using the AUC for discrimination and the Brier Score, calibration-in-the-large, logistic calibration intercept, calibration slope, observed/expected ratio, and expected calibration error for calibration assessment [36]. Confidence intervals were reported from 2000 survey-weighted bootstrap repetitions [37]. For BRFSS evaluation cohorts, resampling was stratified using the retained design fields and survey weights were re-normalized within each replicate. Because the retained BRFSS PSU field was effectively respondent-level within strata, these intervals represent stratified respondent resampling rather than full PSU-cluster resampling. The trained 2016 EBM predictions were held fixed during resampling; therefore, the intervals reflect evaluation-sample uncertainty rather than uncertainty from model retraining or repeated imputation. For the pooled NHANES reference cohort, bootstrap resampling was performed within NHANES cycle with survey-weight re-normalization before metric computation. Pooled cohort-level summaries were generated from survey/cycle-specific weighted estimates rather than by pooling un-normalized individual weights across all years.

#### 2.3.2. Exploratory Cross-Survey Temporal Comparison

We compared two observed periods: BRFSS 2016 versus 2018 and NHANES 2015–2016 versus 2017–2018. Time was coded 0 for the earlier period and 1 for the later period; SurveyType was coded 0 for BRFSS and 1 for NHANES. Weighted Least Squares regression was fitted to individual cross-entropy log-loss, with survey weights normalized to unit mean within each survey–period cell.

The survey-system-by-period interaction model was specified as follows:

*LogLoss_i_* = *β*_0_ + *β*_1_*Time_i_* + *β*_2_*SurveyType_i_* + *β*_3_(*Time_i_*
*×*
*SurveyType_i_*) + *ε_i_*

The interaction beta3 represents an exploratory survey-system-by-period contrast in prediction error. Multiple comparable pre-intervention observations were unavailable, and the parallel-trends assumption could not be directly evaluated. Differences in sampling frames, population composition, survey administration, outcome ascertainment, and third-molar handling also remain inseparable. The coefficient was therefore interpreted as associative rather than as a causal survey-mode effect or a decomposition of epidemiological and measurement processes.

To evaluate whether structurally absent or weakly harmonized predictors influenced the BRFSS–NHANES comparison, we repeated the cross-survey analysis using a reduced harmonized predictor set. This sensitivity model excluded Income, Cost_Barrier, Smoking_Status, and Binge_Drinking because these variables were structurally absent, differentially missing, or not directly comparable between BRFSS and NHANES. The reduced model retained 15 predictors: Age, Sex, Marital, Education, Insurance, General_Health, BMI, Diabetes, Heart_Attack, Stroke, Asthma, Arthritis, Cancer, Kidney, and COPD.

#### 2.3.3. Predictor-Level Shape-Function Stability Testing

Shape functions from EBMs trained independently on the 2016 and 2024 cohorts were compared using age-stratified Monte Carlo permutation testing (B = 2000 per predictor) [38]. The supremum norm [39] was used to detect localized structural differences. Four focal features were selected for interpretation: household income, smoking status, BMI, and age. No multiplicity correction across these four tests was prespecified or applied; their *p*-values are therefore nominal and exploratory. Results for all 19 predictors are provided in Supplementary Table S3.

The global supremum statistic was defined as follows:

*T* = max*_i_* |*f*_2024_(*x_i_*) − *f*_2016_(*x_i_*)|

A sensitivity analysis examined eight BRFSS income tiers anchored to the 2024 Federal Poverty Level (FPL) guidelines [40] to assess whether income-related risk divergence followed a graded relationship (Supplementary Table S4).

For the cumulative income-threshold sensitivity analysis, the threshold-specific supremum statistic was defined as follows:

*T_k_* = max*_i_*_≤_*_k_* |*f*_2024_(*x_i_*) − *f*_2016_(*x_i_*)|

### 2.4. Computational Reproducibility

All analyses were performed using Python 3.11 and InterpretML v0.6.2 [35] with fixed global random seeds to ensure computational reproducibility. Discrimination and calibration metrics incorporated normalized complex survey weights [41]. The complete analytical codebase, including data preprocessing scripts and trained Explainable Boosting Machine models, has been permanently archived on Zenodo and is publicly available at https://doi.org/10.5281/zenodo.19496921.

## 3. Results

### 3.1. Study Population and Baseline Characteristics

Across five BRFSS cycles, the analytic cohort comprised 2,176,039 respondents; pooled NHANES comprised 10,775 participants. Between 2016 and 2024, college education increased from 36.7% to 42.3%, current smoking declined from 14.6% to 10.8%, and severe tooth loss declined from 20.2% to 16.3%. A survey-weighted logistic regression confirmed a decline in severe tooth loss across BRFSS cycles (odds ratio per 2-year increment, 0.960; 95% CI, 0.958 to 0.962; *p* < 0.001). Supplementary Table S5 reports the full descriptive comparison.

### 3.2. Baseline Model Performance and Calibration

The baseline EBM achieved an AUC of 0.8638 (95% CI, 0.8590 to 0.8697) and a Brier Score of 0.0936. Isotonic recalibration produced negligible improvement, with an AUC of 0.8621 and a Brier Score of 0.0938. Therefore, the unrecalibrated baseline EBM was retained for subsequent temporal validation and structural-stability auditing to preserve direct accessibility and comparability of the model-derived shape functions. Comparator-model performance and fixed-threshold classification metrics are reported in Supplementary Table S10.

In the interaction-enabled sensitivity analysis, the 2016 hold-out AUC increased from 0.863801 to 0.864788 (delta = 0.000987; 95% CI, 0.000242 to 0.001807), and Brier Score decreased from 0.093568 to 0.093324 (delta = −0.000244; 95% CI, −0.000453 to −0.000030). In the 2024 hold-out, AUC increased from 0.849539 to 0.851143 (delta = 0.001604; 95% CI, 0.000692 to 0.002428), whereas the Brier difference was negligible (delta = −0.000070; 95% CI, −0.000285 to 0.000152) (Supplementary Table S11).

### 3.3. Longitudinal Temporal Drift Evaluation

**Figure 2:**
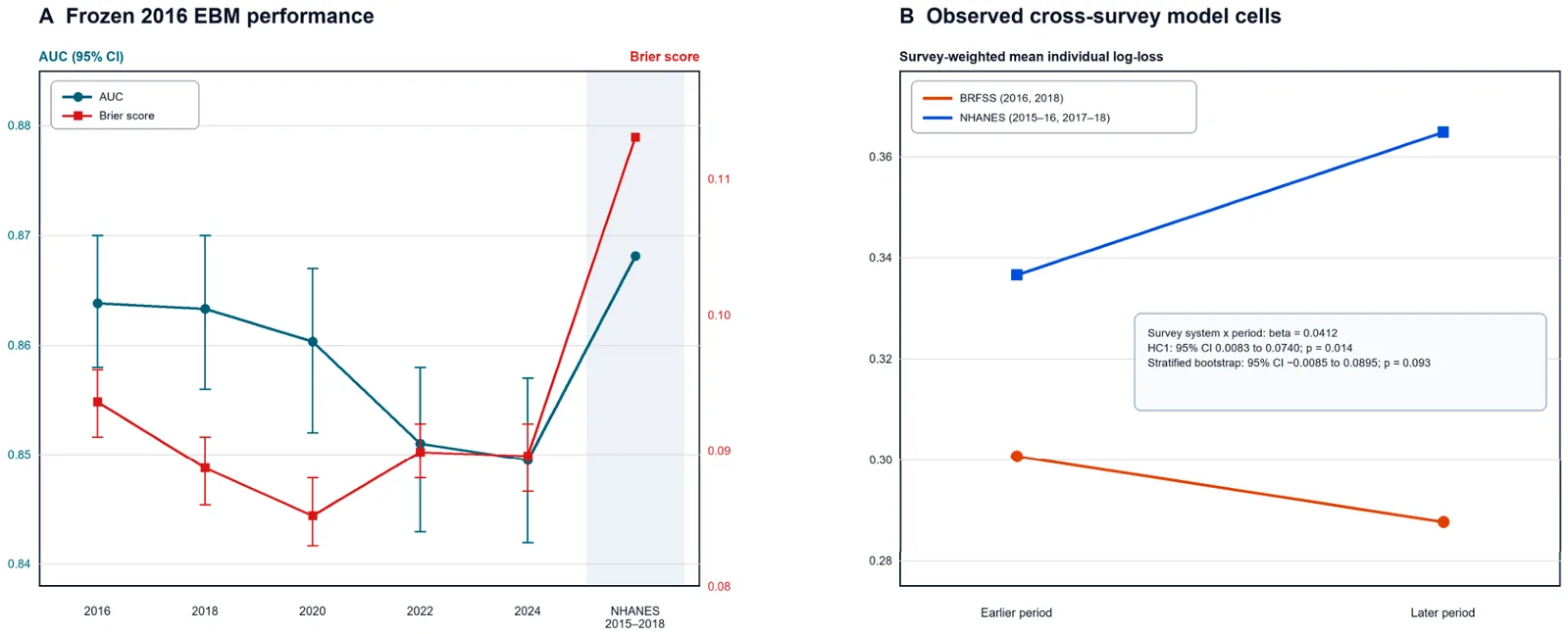
Frozen-model performance and exploratory cross-survey temporal comparison. (**A**) Survey-weighted AUC and Brier Score for the frozen 2016 EBM across BRFSS hold-outs; the pooled NHANES values are unrecalibrated external-reference point estimates. (**B**) The four observed survey–period mean individual log-loss cells used in the interaction model. No counterfactual trajectory is displayed. The interaction estimate was beta3 = 0.0412. The original HC1 interval excluded zero (95% CI, 0.0083 to 0.0740; *p* = 0.014), whereas the stratified survey-weighted bootstrap sensitivity interval included zero (95% CI, −0.0085 to 0.0895; *p* = 0.093).

The 2016 baseline EBM was applied chronologically to hold-out BRFSS cohorts from 2018 through 2024 (Figure 2A). Within-BRFSS discrimination declined modestly over time, with the AUC decreasing from 0.8638 in 2016 to 0.8495 in 2024. The Brier Score decreased transiently in 2020 to 0.0852, before increasing to 0.0896 in 2024. Expanded calibration metrics showed that calibration instability was more apparent than discrimination change alone: the observed/expected ratio declined from 1.0061 in BRFSS 2016 to 0.7893 in BRFSS 2024, CITL shifted from 0.0097 to −0.3645, and ECE increased from 0.0069 to 0.0352. All reported performance and calibration estimates were calculated using cycle-specific normalized survey weights, and uncertainty intervals were obtained from the survey-weighted bootstrap reported in Supplementary Table S6. This transient decrease may be consistent with COVID-19-related changes in dental care utilization and surveillance patterns.

For the primary like-for-like external assessment, the frozen unrecalibrated 2016 EBM was applied to pooled NHANES and yielded AUC = 0.8681, Brier Score = 0.1131, observed/expected ratio = 1.3510, CITL = 0.4740, calibration slope = 1.1949, and ECE = 0.0550 (Supplementary Table S7). Isotonic recalibration was not fitted on NHANES outcomes: the weighted isotonic map was fitted on the BRFSS 2016 hold-out set (N = 94,976) and then applied to NHANES (N = 10,775), yielding AUC = 0.8669 and Brier Score = 0.1124. This recalibrated result is reported only as a sensitivity analysis and is not compared directly with the unrecalibrated BRFSS baseline. The cross-survey differences reflect outcome prevalence, discrimination, calibration, measurement, and population differences rather than calibration alone.

### 3.4. Results of the Exploratory Cross-Survey Temporal Comparison

The survey-system-by-period interaction was positive (beta3 = 0.0412). In the original WLS-HC1 analysis, SE = 0.0168, 95% CI = 0.0083 to 0.0740, and *p* = 0.014. A post hoc 2000-replicate stratified survey-weighted bootstrap sensitivity retained the same point estimate but increased uncertainty (SE = 0.0251; percentile 95% CI, −0.0085 to 0.0895; *p* = 0.093). Thus, the magnitude and direction were stable, whereas statistical significance was sensitive to the uncertainty estimator (Figure 2B; Supplementary Table S8). The reduced harmonized-variable analysis was directionally consistent (beta = 0.0401; HC1 95% CI, 0.0053 to 0.0748; *p* = 0.0239; Supplementary Table S9).

The interaction is an absolute contrast of 0.0412 log-loss units. Relative to the survey-weighted BRFSS 2016 reference-cell mean log-loss of 0.3007, it corresponds to 13.69%; this ratio is scale- and reference-dependent and is not a percentage-point change in individual risk. The contrast combines survey administration, sampling, population, predictor harmonization, and outcome-ascertainment differences and should not be interpreted as a pure survey-mode effect.

The cross-survey comparison identified a larger prediction-error discrepancy than the within-BRFSS AUC trajectory alone. Methodologically independent reference cohorts may therefore add a useful monitoring signal, provided that differences in measurement, design, and population are reported and the comparison is not given a causal interpretation.

### 3.5. Permutation Testing and Predictor-Level Structural Stability

Shape functions from independently trained 2016 and 2024 BRFSS EBMs were compared using age-stratified permutation testing (Table 2, Panel A). Smoking status showed the largest nominal shift (delta = 0.4312; *p* < 0.001), and age also shifted (delta = 0.3855; *p* < 0.001). BMI did not show a detectable shift (delta = 0.1042; *p* = 0.369), indicating that the procedure did not uniformly flag all predictors. These are predictor-level structural results, not group-based fairness metrics.

**Figure 3:**
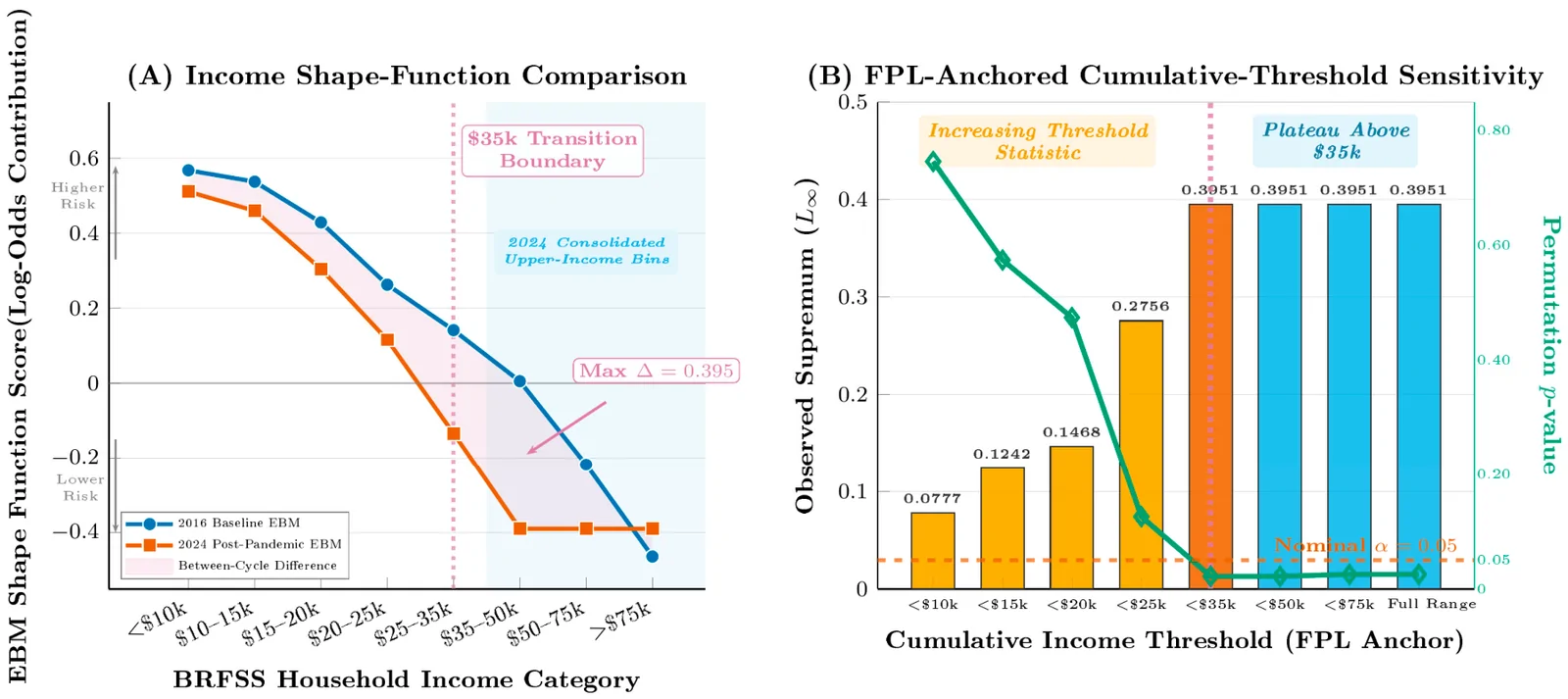
Income-related structural change in EBM risk functions and Federal Poverty Level (FPL) sensitivity analysis. (**A**) EBM income shape functions in 2016 and 2024. The 2024 function shows a threshold-like transition between $25,000 and $35,000 compared with the more gradual 2016 baseline pattern. The vertical dashed line indicates the main income transition boundary, and the shaded region indicates the consolidated upper-income risk pattern. The maximum observed shape-function difference was 0.395. (**B**) Sensitivity analysis across cumulative 2024 FPL thresholds. Bars indicate the observed supremum statistic on the left axis, and the line indicates permutation *p*-values on the right axis. The supremum statistic increased across lower-income thresholds, peaked at the $35,000 boundary, and then plateaued at higher thresholds.

Household income showed a nominal temporal shift (delta = 0.3951; *p* = 0.030). The cumulative-threshold sensitivity increased from a supremum statistic of 0.0777 below $10,000 to 0.3951 below $35,000, where the nominal *p*-value was 0.022, and then plateaued at higher thresholds. These threshold tests would not survive strict family-wise correction and are interpreted as exploratory evidence of an income-related structural pattern rather than definitive confirmation (Figure 3). The finding does not by itself demonstrate inequity or deterioration in algorithmic fairness.

## 4. Discussion

This study evaluates algorithmovigilance as a retrospective quality-monitoring function for population-level oral-health prediction. The central finding is that modest change in aggregate discrimination can coexist with calibration differences and predictor-specific instability. Combining longitudinal performance surveillance, an exploratory cross-survey comparison, and an interpretable EBM shape-function monitoring provides signals that can support governed review before implementation. This emphasis on connecting quantitative alerts with a multidisciplinary review is consistent with recent clinical-AI monitoring research [14,15].

A methodological strength is the use of model-intrinsic shape functions that are directly comparable across cohorts [20,35]. The interaction-enabled sensitivity also quantified the cost of this interpretability choice: AUC improved by only 0.0010 in the 2016 hold-out and 0.0016 in 2024, while the 2024 Brier difference was negligible. Main effects were therefore retained for the primary structural analysis, with the small predictive trade-off reported explicitly.

The reported AUC, log-loss, and supremum differences should be interpreted as study-specific review signals rather than automatic recalibration or retirement thresholds. Operational use would require local data mapping, prospective validation, threshold selection, governance review, and impact evaluation. Transferability to other outcomes, architectures, countries, or clinical settings remains to be established.

A practical deployment workflow would periodically calculate locally validated metrics and route exceeded thresholds to a multidisciplinary governance group for contextual review. Decision Curve Analysis was not added because this retrospective surveillance study does not define a treatment, clinical action, or defensible threshold-probability range; decision utility should be evaluated prospectively once an intended action and its consequences are specified.

The cross-survey interaction should be interpreted cautiously. Its positive magnitude was unchanged in the stratified survey-weighted bootstrap sensitivity, but the wider interval included zero. Moreover, BRFSS and NHANES differ in sampling, administration, population composition, and outcome ascertainment. The analysis therefore provides an exploratory survey-system-by-period signal, not a causal decomposition of epidemiological and survey-mode effects.

Income and smoking showed the largest predictor-level structural changes, while BMI remained comparatively stable. The income *p*-value was nominal and did not meet a four-test Bonferroni threshold, and the threshold-specific analysis was exploratory. Shape-function changes may identify risk mappings that warrant review, but they do not establish group-level unfairness. Equal opportunity, equalized odds, demographic parity, subgroup error rates, and subgroup calibration were not evaluated.

Several contemporaneous factors could contribute to the observed predictor changes, including declining smoking prevalence, pandemic-related disruption of preventive dental care, and changes in healthcare access [42,43]. These explanations were not directly tested and should be regarded as hypotheses rather than identified mechanisms.

The three-tier framework aligns conceptually with lifecycle-monitoring priorities in FDA, WHO, and EU guidance [16,17,18]. The numerical signals observed here are not portable operational thresholds; local prospective validation is required before they can guide decisions.

**Figure 4:**
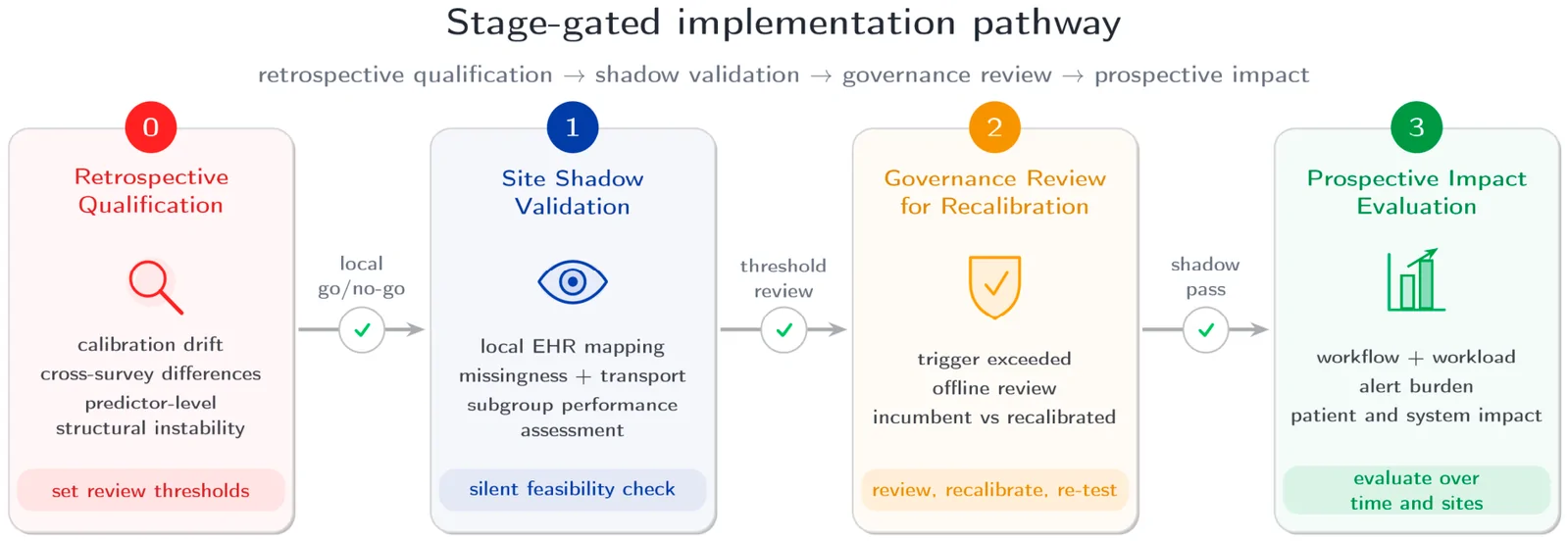
Stage-gated implementation roadmap for future evaluation of the algorithmovigilance framework in AI-based oral-health risk monitoring. The proposed roadmap separates retrospective qualification from future local validation, governance review, and prospective impact evaluation. Stage 0, Retrospective Qualification: Establishes baseline calibration drift, cross-survey differences, and predictor-level structural instability using historical data. This stage represents the empirical contribution of the present study. Stage 1, Site Shadow Validation: Involves silent execution on local oral health, public-health, or surveillance datasets to assess feasibility, data mapping, calibration transport, and subgroup performance assessment before operational use. Stage 2, Governance Review: Involves offline review and candidate recalibration when monitoring signals exceed locally validated triggers, while avoiding autonomous model replacement. Stage 3, Prospective Impact Evaluation: Assesses workflow consequences, equity impact, population-level benefit or harm, workforce burden, and accountability processes after implementation planning.

Figure 4 summarizes a stage-gated pathway from retrospective qualification to site-specific shadow validation, governance review, and prospective impact evaluation. Only retrospective qualification was evaluated in this study; the later stages remain future implementation steps.

The predictor set contains sociodemographic, behavioral, and systemic-health survey variables but no periodontal, oral-clinical, or imaging inputs. This limits clinical granularity and precludes chairside diagnostic claims. Future clinical validation should examine whether adding oral examination and imaging variables improves transportability and decision utility.

Severe tooth loss is etiologically heterogeneous and may reflect periodontal disease, caries, trauma, or combinations of these processes. Because BRFSS and NHANES do not identify the cause of each lost tooth, predictor changes represent shifts in the aggregate risk landscape rather than a single disease pathway.

Additional limitations include self-reported BRFSS tooth loss, examination-based NHANES ascertainment, and non-equivalent third-molar handling [44]. The two surveys are not exchangeable, and multiple comparable pre-period observations were unavailable for evaluating parallel trends. HC1 does not account explicitly for survey clustering and stratification; the added bootstrap sensitivity widened the interaction interval to include zero, and the retained BRFSS PSU field was effectively respondent-level within strata. The four focal permutation tests used nominal *p*-values without a prespecified multiplicity correction, and the income threshold findings remain exploratory. Traditional group-based fairness metrics and sex-, race/ethnicity-, education-, or geography-stratified performance were not evaluated. Finally, this single-country, single-outcome retrospective study has not been tested in clinical or public-health workflows.

## 5. Conclusions

This proof-of-concept study operationalized a retrospective algorithmovigilance workflow for population-level severe tooth loss prediction. A frozen 2016 EBM retained broadly stable discrimination through 2024 while showing calibration and predictor-level structural changes that aggregate AUC alone did not capture. The exploratory BRFSS–NHANES interaction remained positive in magnitude, although its statistical significance was sensitive to the uncertainty estimator. An interaction-enabled EBM produced only small discrimination gains, supporting retention of the main-effects model for transparent temporal comparison. Practically, these outputs should be treated as review signals rather than automated deployment thresholds. The next steps are local shadow validation, formal subgroup fairness assessment, decision-utility analysis tied to a defined action, and prospective evaluation of clinical and population-level impact.

## Figures and Tables

**Table 1:** Predictors, harmonized coding, and pre-imputation missingness in BRFSS.

Predictor	Coding Used in Model	2016 Baseline Missingness (%)	Pooled BRFSS Missingness 2016–2024 (%)
Age	_AGEG5YR	1.290	1.721
Sex	SEXVAR/SEX1/SEX	0.011	0.046
Marital status	MARITAL	0.629	0.809
Education	_EDUCAG	0.318	0.396
Income	_INCOMG1/INCOME3/INCOME2	16.229	18.393
Health insurance	_HLTHPLN1/_HLTHPLN/_HLTHPL2	0.367	1.811
Cost barrier for medical care	MEDCOST1/MEDCOST	0.241	0.316
General health	GENHLTH	0.257	0.240
BMI	_BMI5	7.912	9.094
Smoking status	_SMOKER3	3.963	5.490
Binge drinking	_RFBING6/_RFBING5	5.819	8.122
Diabetes	DIABETE4/DIABETE3	0.151	0.173
Heart attack	CVDINFR4	0.446	0.526
Stroke	CVDSTRK3	0.254	0.273
Asthma	ASTHMA3	0.272	0.317
Arthritis	HAVARTH4/HAVARTH3	0.471	0.512
Cancer	CHCOCNC1/CHCOCNCR	0.216	0.337
Kidney disease	CHCKDNY2/CHCKDNY1/CHCKIDNY	0.299	0.347
COPD	CHCCOPD3/CHCCOPD2/CHCCOPD1	0.420	0.435

Note: Sex: 2016 = SEX, 2018 = SEX1, 2020–2024 = SEXVAR; 2016–2020 = INCOME2, 2022–2024 = _INCOMG1; 2016–2020 = _HLTHPLN1, 2022 = _HLTHPLN, 2024 = _HLTHPL2; 2016–2020 = MEDCOST, 2022–2024 = MEDCOST1; 2016–2020 = _RFBING5, 2022–2024 = _RFBING6; 2016–2018 = DIABETE3, 2020–2024 = DIABETE4; 2016–2018 = HAVARTH3, 2020–2024 = HAVARTH4; 2016–2020 = CHCOCNCR, 2022–2024 = CHCOCNC1; 2016 = CHCKIDNY, 2018 = CHCKDNY1, 2020–2024 = CHCKDNY2; 2016–2018 = CHCCOPD1, 2020 = CHCCOPD2, 2022–2024 = CHCCOPD3.

**Table 2:** Supremum-norm permutation test results and Federal Poverty Level-based sensitivity analysis.

**Panel A. Permutation Test Results for Temporal Changes in Predictor-Specific Risk Patterns (Age-Stratified, B = 2000 Permutations)**				
**Feature**	**Observed Supremum Statistic** **(**$L_{\infty }$**)**	***p*-Value**	**Interpretation**	
Smoking status	0.4312	<0.001	Significant temporal shift	
Age	0.3855	<0.001	Significant temporal shift	
Income	0.3951	0.030	Nominal temporal shift; exploratory	
BMI	0.1042	0.369	Not statistically significant	
**Panel B. Income Sensitivity Analysis Based on Federal Poverty Level Thresholds (B = 2000 Permutations)**				
**Household Income Threshold**	**FPL** **Reference**	**Cumulative N** **(2016/2024)**	**Observed Supremum Statistic** **(**$L_{\infty }$**)**	***p*** **-Value**
<$10,000	Approximately 67% of FPL for a 1-person household	19,315/20,325	0.0777	0.747
<$15,000	100% of FPL for a 1-person household	42,066/58,403	0.1242	0.574
<$20,000	Approximately 100% of FPL for a 2-person household	82,058/118,302	0.1468	0.475
<$25,000	Approximately 100% of FPL for a 3-person household	140,374/196,683	0.2756	0.125
<$35,000	100% of FPL for a 4-person household	213,666/348,215	0.3951	0.022
<$50,000 *	Higher-income threshold	303,555/448,213	0.3951	0.023
<$75,000 *	Higher-income threshold	333,961/448,213	0.3951	0.025
Full Range *	All income strata	474,879/448,213	0.3951	0.025

Note: BMI, body mass index; FPL, Federal Poverty Level. Panel A reports nominal permutation tests for four focal features; no multiplicity correction across these tests was prespecified or applied. Panel B evaluates cumulative income thresholds, with higher-income cutoffs treated as exploratory. Under a four-test Bonferroni threshold of 0.0125, the nominal income *p*-value of 0.030 would not be significant. The threshold-specific results are hypothesis-generating. * Indicates the exploratory higher-income thresholds (≤$50,000 and ≤$75,000) and the full-range comparison; these rows do not correspond to a single FPL reference threshold.

## Data Availability

The BRFSS and NHANES datasets used in this study are publicly available from the Centers for Disease Control and Prevention at https://www.cdc.gov/brfss/ (accessed on 12 June 2026) and https://www.cdc.gov/nchs/nhanes/ (accessed on 2 May 2026), respectively. The complete analytical codebase, including data preprocessing scripts and trained Explainable Boosting Machine models, has been permanently archived on Zenodo and is publicly available at https://doi.org/10.5281/zenodo.19496921.

## Supplementary Materials

The following supporting information can be downloaded at: https://www.mdpi.com/article/10.3390/diagnostics16162569/s1, Table S1: Completed TRIPOD+AI Checklist; Table S2: Hyperparameter Optimization Grid for the Baseline Explainable Boosting Machine (EBM); Table S3: Permutation Test Results for All 19 EBM Predictors; Table S4: Mapping of BRFSS Categorical Income Tiers against the 2024 U.S. Federal Poverty Level (FPL) Guidelines; Table S5: Demographic and clinical characteristics of the BRFSS and NHANES study populations; Table S6: Survey-weighted bootstrap confidence intervals for primary discrimination and calibration metrics; Table S7: Expanded calibration metrics for the frozen unrecalibrated primary 19-feature EBM across BRFSS cycles and pooled NHANES; Table S8: Weighted Least Squares exploratory survey-system-by-period interaction in individual log-loss; Table S9: Reduced harmonized-variable sensitivity analysis excluding structurally absent or weakly harmonized predictors across BRFSS and NHANES; Table S10: Comparator model performance and fixed-threshold classification metrics for EBM, XGBoost, and LightGBM across BRFSS temporal validation cohorts and pooled NHANES; Table S11: Interaction-enabled EBM sensitivity analysis.
