# Supplementary material for: Algorithmovigilance in AI-Based Oral-Health Surveillance: Temporal Drift, Cross-Survey Differences, and Predictor-Level Structural Stability in Population-Level Severe Tooth Loss Prediction
Source: Diagnostics (Basel). 2026 Aug 14;16(16):2569. doi: 10.3390/diagnostics16162569 (PMC13512040; doi:10.3390/diagnostics16162569)
Supplement: Supplementary file 1 [file diagnostics-16-02569-s001.zip › diagnostics-4471218-supplementary.pdf]

SUPPLEMENTARY MATERIALS

Supplementary Table S1: Completed TRIPOD+AI checklist

Transparent Reporting of a multivariable prediction model of Individual Prognosis Or Diagnosis paired with Artificial Intelligence (TRIPOD+AI) statement (2024)

Item No.	Section / Topic	Checklist Item	Location in Manuscript
1	Title	Identify the study as developing or evaluating the performance of a multivariable prediction model, specify target population, and identify outcome.	Title Page
2	Abstract	Follow the TRIPOD+AI for Abstracts checklist.	Abstract
3	Background	Explain the medical context, including whether the prediction model is diagnostic or prognostic.	Introduction, Paragraphs 1–3
4	Objectives	Specify objectives for model evaluation, temporal monitoring, and predictor-level structural analysis.	Introduction, Final Paragraph
5	Study Design	State the study design or source of data (e.g., longitudinal survey, registry, cohort).	Section 2.1 Dataset, Study Population, and Variable Operationalization
6	Setting	Describe the setting, locations, and relevant dates (e.g., periods of recruitment, exposure, follow-up).	Section 2.1 Dataset, Study Population, and Variable Operationalization
7	Participants	Describe eligibility criteria for participants and sources of predictor and outcome data.	Section 2.1 Dataset, Study Population, and Variable Operationalization
8	Outcome	Clearly define the outcome(s) being predicted and how/when it was assessed.	Section 2.1 Dataset, Study Population, and Variable Operationalization
9	Predictors	List the predictors used, and describe how/when they were measured.	Section 2.1 and Table 1
10	Sample Size	Explain how the study size was arrived at to ensure reliable model development or evaluation.	Section 2.1 and Section 3.1 Study Population and Baseline Characteristics
11	Missing Data	Describe how missing data for predictors and outcomes were handled (e.g., imputation methods).	Section 2.2 Missing Data Imputation and Explainable Boosting Machine; Table 1

Item No.	Section / Topic	Checklist Item	Location in Manuscript
12	Model & AI Architecture	Describe the statistical/machine learning modeling methods used (Specify algorithms, e.g., Explainable Boosting Machine).	Section 2.2; Supplementary Table S2
13	Hyperparameters	Specify all data preprocessing steps, hyperparameter tuning, and internal validation procedures.	Section 2.2; Supplementary Table S2
14	Performance Evaluation	Specify the criteria and metrics used to evaluate model performance (e.g., AUC, Brier).	Section 2.3.1 Temporal Drift Evaluation; Supplementary Tables S6–S7
15	Structural Stability	Describe predictor-level structural stability methods and distinguish them from formal group-based fairness metrics.	Section 2.3.3; Table 2; Supplementary Table S3
16	Hardware/Software	Provide details on the computer hardware, software packages, and versions used.	Section 2.4 Computational Reproducibility
17	Participants (Results)	Describe the flow of participants through the study, including numbers with and without the outcome.	Section 3.1 Study Population and Baseline Characteristics; Supplementary Table S5
18	Characteristics	Describe the characteristics of the participants (demographic, clinical, predictors).	Section 3.1; Table 1; Supplementary Table S5
19	Model Specification	For AI models, describe how the final model is presented (e.g., shape functions, feature importance).	Section 2.2; Section 3.5; Figure 3; Supplementary Table S2
20	Model Performance	Report performance measures (with confidence intervals) for the evaluation dataset.	Sections 3.2–3.3; Figure 2; Supplementary Tables S6–S7
21	Updating/Drift	Report the temporal performance changes or any model updating performed.	Sections 3.3–3.4; Figure 2; Supplementary Table S8
22	Structural Stability Results	Report predictor-level structural findings and their exploratory interpretation.	Section 3.5; Table 2; Figure 3; Supplementary Tables S3–S4
23	Limitations	Discuss limitations of the study (e.g., study design, missing data, applicability).	Discussion, Limitations Paragraph

Item No.	Section / Topic	Checklist Item	Location in Manuscript
24	Interpretation	Give an overall interpretation of the results considering objectives, limitations, and similar studies.	Discussion, Opening and Interpretation Paragraphs
25	Implications	Discuss the potential clinical utility and implications of the model/findings.	Discussion, Governance/Roadmap Paragraph; Figure 4
26	Open Science	Provide information on the availability of study protocol, data, models, and code.	Data Availability Statement; Section 2.4 Computational Reproducibility
27	Funding / COI	Provide details of funding and any conflicts of interest.	Funding; Conflicts of Interest

Supplementary Table S2: Hyperparameter optimization grid for the baseline Explainable Boosting Machine (EBM)

Item	Value	Notes
Model	ExplainableBoostingClassifier	Baseline additive EBM
Training cohort	BRFSS 2016	19 predictors
Outcome	Severe_Tooth_Loss	Binary target
Predictors	19	Age, Sex, Marital, Education, Income, Insurance, Cost_Barrier, General_Health, BMI, Smoking_Status, Binge_Drinking, Diabetes, Heart_Attack, Stroke, Asthma, Arthritis, Cancer, Kidney, COPD
Sample weights	Survey weights normalized to sample size	Native weighted fitting
Objective	log_loss	Model default
Interactions	0	Main effects only
Learning rate	0.01	Model default
Max bins	1024	Model default
Max leaves	3	Model default
Max rounds	25000	Upper bound; early stopping applied
Outer bags	14	Model default
Inner bags	0	Model default
Validation size	0.15	Internal early-stopping split
Early stopping rounds	50	Model default
Smoothing rounds	200	Model default
Interaction smoothing rounds	50	Model default
Greedy ratio	1.5	Model default
Min samples leaf	2	Model default

Min hessian	0.0001	Model default
Cyclic progress	True	Model default
Random state	42	Fixed seed
Explicit L1/L2 regularization	Not used	EBM complexity controlled by binning, leaf count, smoothing, and early stopping
Cross-validation for final reported model	Not used	Final temporal evaluation was chronological hold-out across BRFSS years

Note: The baseline model was constrained to main effects to preserve direct univariate interpretability and temporal comparability. A like-for-like interaction-enabled sensitivity analysis is reported in Supplementary Table S11.

Supplementary Table S3: Permutation test results for all 19 EBM predictors

Predictor	Observed supremum statistic (L_∞)	Permutation p-value
Age	0.3855	$p < 0.001$
Smoking_Status	0.4312	$p < 0.001$
Income	0.3951	$p = 0.030$
BMI	0.1042	$p = 0.369$
Cancer	0.1335	$p = 0.425$
Arthritis	0.0687	$p = 0.465$
Education	0.1071	$p = 0.662$
Sex	0.0244	$p = 0.675$
COPD	0.0648	$p = 0.692$
Kidney	0.0827	$p = 0.703$
Insurance	0.0657	$p = 0.721$
Binge_Drinking	0.050	$p = 0.760$
Asthma	0.0335	$p = 0.778$
Stroke	0.0444	$p = 0.824$
Cost_Barrier	0.0243	$p = 0.881$
General_Health	0.0770	$p = 0.896$
Diabetes	0.1598	$p = 0.923$
Heart_Attack	0.0199	$p = 0.929$
Marital	0.0906	$p = 0.951$

Note. Age, Smoking_Status, and Income showed nominal significance in this all-predictor transparency analysis. Four focal predictors were emphasized in the manuscript, but no multiplicity correction across those four tests was prespecified or applied; the p-values are interpreted as exploratory.

Supplementary Table S4: Mapping of BRFSS categorical income tiers against the 2024 U.S. Federal Poverty Level (FPL) Guidelines

BRFSS Tier	Annual Household Income Bracket	Contextual 2024 HHS FPL alignment (contiguous U.S.)
1	Less than $10,000	Below the one-person guideline ($15,060)
2	$10,000 to < $15,000	Below the one-person guideline ($15,060)
3	$15,000 to < $20,000	Spans the one-person guideline and remains below the two-person guideline ($20,440)
4	$20,000 to < $25,000	Spans the two-person guideline and remains below the three-person guideline ($25,820)
5	$25,000 to < $35,000	Spans the three-person ($25,820) and four-person ($31,200) guidelines
6	$35,000 to < $50,000	Above the four-person guideline ($31,200)
7	$50,000 to < $75,000	Above the four-person guideline ($31,200)
8	$75,000 or more	Above the four-person guideline ($31,200)

Note: BRFSS assesses annual household income using eight categorical tiers. The absolute dollar intervals are shown alongside the 2024 U.S. Department of Health and Human Services Poverty Guidelines for contextual mapping only. No tier-specific inferential tests are reported in this table; cumulative-threshold permutation results are reported in main Table 2 and are exploratory.

Source: Department of Health and Human Services. Annual Update of the HHS Poverty Guidelines. *Federal Register*. January 17, 2024;89(11):2961-2963.

Supplementary Table S5: Demographic and clinical characteristics of the BRFSS and NHANES study populations

Characteristic	BRFSS (2016-2024), unweighted descriptive (N = 2,176,039)	NHANES (2015-2018), unweighted descriptive (N = 10,775)	BRFSS Missingness (%)	NHANES Missingness (%)
**Primary outcome**				
Severe tooth loss (≥6 teeth lost), %	17.8%	27.0%	0.0%	0.0%
**Demographic characteristics**				
Age, mean ± SD, years*	54.9 ± 19.9	48.9 ± 18.6	0.0%**	0.0%
Sex, % female / male	54.1% / 45.9%	51.6% / 48.4%	0.0%	0.0%
Married, %	51.9%	50.1%	0.8%	4.8%
**Race and ethnicity, %**				
Non-Hispanic White	74.2%	—	0.0%***	< 0.1%
Hispanic	9.2%	—		
Non-Hispanic Black	7.9%	—		
Other or multiracial	6.7%	—		
**Socioeconomic characteristics**				
College graduate or higher, %	39.8%	24.6%	0.4%	4.8%
Household income, 8 categories	—	—	28.8%	11.7%
**Healthcare access**				
Health insurance coverage, %	93.0%	83.5%	1.8%	0.0%
Cost barrier to medical care, % yes	9.5%	Not surveyed	0.3%	100.0%
**Health behaviors**				
Self-rated general health, % good to excellent	81.8%	75.0%	0.2%	0.0%
BMI, mean ± SD, kg/m²	28.3 ± 6.4	29.5 ± 7.3	9.1%	1.3%
Never smoked, %	58.7%	59.6%	5.5%	0.0%
Binge drinking, % yes	13.6%	Not surveyed	8.1%	58.8%****
**Chronic conditions**				
Diabetes, %	13.6%	14.5%	0.2%	0.0%
History of heart attack, %	5.8%	4.6%	0.5%	4.8%
History of stroke, %	4.2%	4.2%	0.3%	4.8%
Asthma, %	14.4%	15.4%	0.3%	0.0%
Arthritis, %	33.7%	28.4%	0.5%	4.8%
Cancer, %	10.4%	10.1%	0.3%	4.8%
Kidney disease, %	4.2%	6.2%	0.3%	4.8%
COPD, %	8.1%	4.3%	0.4%	4.8%

Characteristic	BRFSS (2016-2024), unweighted descriptive (N = 2,176,039)	NHANES (2015-2018), unweighted descriptive (N = 10,775)	BRFSS Missingness (%)	NHANES Missingness (%)
**Survey modality**				
Cellular telephone interview, %	68.0%	Not applicable	0.0%	Not applicable
Landline telephone interview, %	32.0%	Not applicable	0.0%	Not applicable

*Continuous values were approximated using CDC bracket midpoints. Percentages and means in this table are unweighted descriptive summaries. **No structural missingness. ***Race and ethnicity characteristic was reported descriptively and was not included as a predictive feature. ****In NHANES, cost barrier to medical care was not surveyed, and binge drinking was affected by inter-cycle methodological differences. Missingness values are reported before imputation. — indicates no summary value; Not surveyed indicates that the variable was not collected; Not applicable indicates that the variable was not relevant to that survey design.

Supplementary Table S6. Survey-weighted bootstrap confidence intervals for primary discrimination and calibration metrics.

Dataset	N	AUC (95% CI)	Brier (95% CI)	CITL (95% CI)	Slope (95% CI)	ECE
BRFSS 2016	94,976	0.864 (0.858-0.870)	0.094 (0.091-0.096)	0.010 (-0.029 to 0.047)	1.028 (0.997-1.061)	0.007
BRFSS 2018	85,403	0.863 (0.856-0.870)	0.089 (0.086-0.091)	-0.108 (-0.149 to -0.067)	1.011 (0.973-1.047)	0.011
BRFSS 2020	78,433	0.860 (0.852-0.867)	0.085 (0.083-0.088)	-0.130 (-0.177 to -0.082)	0.996 (0.954-1.035)	0.011
BRFSS 2022	86,755	0.851 (0.843-0.858)	0.090 (0.088-0.092)	-0.354 (-0.400 to -0.306)	1.031 (0.990-1.070)	0.034
BRFSS 2024	89,643	0.850 (0.842-0.857)	0.090 (0.087-0.092)	-0.365 (-0.407 to -0.319)	1.033 (0.996-1.074)	0.035

Note: CI, confidence interval; CITL, calibration-in-the-large; ECE, expected calibration error. Confidence intervals were reported from 2,000 bootstrap repetitions. For BRFSS, resampling was stratified using the retained design fields and survey weights were re-normalized within each replicate. Because the retained BRFSS PSU field was effectively respondent-level within strata, the BRFSS component represents stratified respondent resampling rather than full PSU-cluster resampling. For pooled NHANES, resampling was performed within NHANES cycle using genuine PSU-within-stratum identifiers, with survey-weight re-normalization before metric computation. ECE is reported as a point estimate.

Supplementary Table S7. Expanded calibration metrics for the frozen unrecalibrated primary 19-feature EBM across BRFSS cycles and pooled NHANES.

Dataset	N	AUC	Brier	Observed	Predicted	O/E	CITL	Slope	ECE	Cal. intercept
BRFSS 2016	94,976	0.864	0.094	0.151	0.150	1.006	0.010	1.028	0.007	0.042
BRFSS 2018	85,403	0.863	0.089	0.138	0.148	0.934	-0.108	1.011	0.011	-0.096
BRFSS 2020	78,433	0.860	0.085	0.129	0.140	0.920	-0.130	0.996	0.011	-0.134
BRFSS 2022	86,755	0.851	0.090	0.134	0.168	0.796	-0.354	1.031	0.034	-0.325
BRFSS 2024	89,643	0.850	0.090	0.132	0.167	0.789	-0.365	1.033	0.035	-0.336
NHANES 2015-2018 pooled	10,775	0.868	0.113	0.192	0.142	1.351	0.474	1.195	0.055	0.754

Note: Isotonic sensitivity—the weighted isotonic map was fitted on the BRFSS 2016 hold-out set (N = 94,976), not on NHANES outcomes, and then applied to pooled NHANES (N = 10,775). The resulting metrics were AUC = 0.8669, Brier Score = 0.1124, observed/expected ratio = 1.3366, CITL = 0.4647, slope = 1.1288, and ECE = 0.0501. This sensitivity is reported separately from the frozen unrecalibrated primary assessment.

Supplementary Table S8: Weighted Least Squares exploratory survey-system-by-period interaction in individual log-loss

Parameter	Coefficient	SE (HC1)	*z* statistic	*p* value	95% CI
Intercept	0.3007	0.004	82.771	< 0.001	[0.294, 0.308]
Time	−0.0130	0.005	−2.534	0.011	[−0.023, −0.003]
Survey type	0.0359	0.011	3.257	0.001	[0.014, 0.058]
Time × survey type	0.0412	0.017	2.454	0.014	[0.0083, 0.0740]

The table reports the original WLS analysis with HC1 heteroskedasticity-robust standard errors. A post hoc 2,000-replicate stratified survey-weighted bootstrap sensitivity retained beta = 0.0412 but yielded SE = 0.0251, percentile 95% CI = -0.0085 to 0.0895, and p = 0.093. HC1 does not account explicitly for PSU clustering or stratification. In the bootstrap sensitivity, the retained BRFSS PSU field was effectively respondent-level within strata, so the BRFSS component represents stratified respondent resampling, whereas NHANES retained genuine PSU-within-stratum resampling. The survey-weighted BRFSS 2016 reference-cell mean individual log-loss was 0.300683; relative to this denominator, the interaction contrast of 0.041165 corresponds to 13.69%. This ratio is scale- and reference-dependent and is not a percentage-point change in individual risk.

Supplementary Table S9. Reduced harmonized-variable sensitivity analysis excluding structurally absent or weakly harmonized predictors across BRFSS and NHANES.

Dataset	N	AUC	Brier	Observed/	CITL	Slope	ECE

				Expected			
BRFSS 2016	94,976	0.8466	0.0978	1.0074	0.0113	1.0332	0.0092
BRFSS 2018	85,403	0.8442	0.0927	0.9177	-0.1303	1.0194	0.0124
BRFSS 2020	78,433	0.8422	0.0891	0.8990	-0.1583	1.0052	0.0145
BRFSS 2022	86,755	0.8370	0.0919	0.8907	-0.1730	1.0026	0.0164
BRFSS 2024	89,643	0.8326	0.0921	0.8676	-0.2108	0.9975	0.0201
NHANES 2015 2018 pooled	10,775	0.8606	0.1173	1.4405	0.5654	1.1728	0.0622

Supplementary Table S10. Comparator model performance and fixed-threshold classification metrics for EBM, XGBoost, and LightGBM across BRFSS temporal validation cohorts and pooled NHANES.

Model	Dataset	N	AUC	Brier	ECE	Sensitivity	Specificity	Precision	F1-score	MCC
EBM	BRFSS 2016	94,976	0.8638	0.0936	0.0069	0.8081	0.7585	0.3724	0.5098	0.4320
EBM	BRFSS 2018	85,403	0.8633	0.0887	0.0112	0.8123	0.7596	0.3515	0.4907	0.4234
EBM	BRFSS 2020	78,433	0.8603	0.0852	0.0112	0.7917	0.7726	0.3394	0.4751	0.4122
EBM	BRFSS 2022	86,755	0.8510	0.0899	0.0342	0.8404	0.6978	0.2999	0.4421	0.3783
EBM	BRFSS 2024	89,643	0.8495	0.0896	0.0352	0.8379	0.6944	0.2937	0.4350	0.3717
EBM	NHANES 2015-2018 pooled	10,775	0.8681	0.1131	0.0550	0.7696	0.7838	0.4576	0.5739	0.4660
XGBoost	BRFSS 2016	94,976	0.8635	0.0936	0.0021	0.8167	0.7478	0.3648	0.5043	0.4271
XGBoost	BRFSS 2018	85,403	0.8648	0.0884	0.0109	0.8239	0.7472	0.3433	0.4847	0.4187
XGBoost	BRFSS 2020	78,433	0.8615	0.0848	0.0113	0.8102	0.7566	0.3295	0.4684	0.4081
XGBoost	BRFSS 2022	86,755	0.8505	0.0909	0.0386	0.8546	0.6735	0.2873	0.4300	0.3670
XGBoost	BRFSS 2024	89,643	0.8480	0.0906	0.0382	0.8517	0.6731	0.2833	0.4251	0.3629
XGBoost	NHANES 2015-2018 pooled	10,775	0.8677	0.1097	0.0330	0.8132	0.7489	0.4342	0.5661	0.4612
LightGBM	BRFSS 2016	94,976	0.8642	0.0935	0.0028	0.8259	0.7403	0.3605	0.5019	0.4260

Model	Dataset	N	AUC	Brier	ECE	Sensitivity	Specificity	Precision	F1-score	MCC
LightGBM	BRFSS 2018	85,403	0.8651	0.0882	0.0091	0.8312	0.7413	0.3401	0.4827	0.4178
LightGBM	BRFSS 2020	78,433	0.8621	0.0847	0.0112	0.8181	0.7484	0.3243	0.4644	0.4051
LightGBM	BRFSS 2022	86,755	0.8492	0.0912	0.0373	0.8565	0.6703	0.2858	0.4286	0.3658
LightGBM	BRFSS 2024	89,643	0.8484	0.0904	0.0372	0.8538	0.6684	0.2809	0.4227	0.3604
LightGBM	NHANES 2015-2018 pooled	10,775	0.8665	0.1097	0.0335	0.8249	0.7260	0.4164	0.5534	0.4468

Note: For EBM rows, ECE was calculated using the same survey-weighted 10-bin prediction-quantile pipeline and frozen unrecalibrated predictions as Supplementary Table S7; the resulting BRFSS 2016 and pooled NHANES values are 0.0069 and 0.0550, respectively. Sensitivity, specificity, precision, F1-score, and MCC use a fixed probability threshold of 0.1507, selected by maximizing survey-weighted Youden's J on the BRFSS 2016 training set and then locked for all hold-out and external cohorts.

Supplementary Table S11. Interaction-enabled EBM sensitivity analysis

Cohort	N	Metric	Main effects	Interactions	Difference	95% CI	p value
BRFSS 2016 hold-out	94,976	AUC	0.863801	0.864788	+0.000987	0.000242 to 0.001807	0.013
BRFSS 2016 hold-out	94,976	Brier	0.093568	0.093324	-0.000244	-0.000453 to -0.000030	0.023
BRFSS 2024 hold-out	89,643	AUC	0.849539	0.851143	+0.001604	0.000692 to 0.002428	0.003
BRFSS 2024 hold-out	89,643	Brier	0.089592	0.089522	-0.000070	-0.000285 to 0.000152	0.522

Note: The interaction-enabled EBM used the same survey-weighted 2016 training set (N = 379,903), 19 predictors, and baseline hyperparameters, except that automatic pairwise interactions were enabled (interactions = 0.9; 18 terms selected). Differences are interaction-enabled minus main-effects. Confidence intervals and p values are from 2,000 paired stratified survey-weighted bootstrap replicates; for BRFSS, the retained PSU field was effectively respondent-level within strata.
